# University indigenous students' perspectives on climate change and survival of indigenous peoples in Brazil: a concept mapping study

**DOI:** 10.3389/fpubh.2023.1236662

**Published:** 2023-11-30

**Authors:** Antonio Jose Grande, Ieda M. A. V. Dias, Paulo T. C. Jardim, Alessandra Aparecida Vieira Machado, Jacks Soratto, Maria Inês da Rosa, Leonardo Roever, Luciane Bisognin Ceretta, Xanthi Zourntos, Seeromanie Harding

**Affiliations:** ^1^Medicine School, State University of Mato Grosso do Sul, Campo Grande, Mato Grosso do Sul, Brazil; ^2^Department of Public Health, Federal University of Rio Grande do Sul, Porto Alegre, Rio Grande do Sul, Brazil; ^3^Public Health Department, Universidade do Extremo Sul Catarinense, Criciuma, Santa Catarina, Brazil; ^4^Gilbert and Rose-Marie Chagoury School of Medicine, Lebanese American University, Beirut, Lebanon; ^5^Department of Population Health Sciences, School of Population Health & Environmental Sciences, Faculty of Life Sciences & Medicine, King's College London, London, United Kingdom

**Keywords:** indigenous, climate change, land loss, survival, health, concept mapping, community based participatory research

## Abstract

**Introduction:**

This study aimed to identify what indigenous university students in Brazil perceived to be important and feasible actions to protect the survival of indigenous peoples from climate change-related impacts.

**Methods:**

Concept mapping, which is a participatory mixed methodology, was conducted virtually with 20 indigenous students at two universities in Brazil. A focus prompt was developed from consultations with indigenous stakeholders and read “*To protect the survival of the Indigenous Peoples from climate change, it is necessary to*…”. Students brainstormed 46 statements, which they then sorted into clusters based on conceptual similarity. They rated each statement for importance and feasibility. Quantitative multivariate analyses of clusters and ratings were conducted to produce multiple visual maps of perceived actionable priorities. These analyses used the Group Wisdom TM software.

**Results:**

Students agreed on 8 clusters that reflect the factors that influence the survival of indigenous peoples-preservation of lands 0.16 (SD 0.13), protection of demarcated lands 0.31 (SD 0.10), indigenous health and wellbeing 0.35 (SD 0.14), ancestral customs 0.46 (SD 0.04), global and national actions 0.61 (SD 0.13), indigenous rights 0.64 (SD 0.23), collective living 0.71 (SD 0.21), and respect 0.75 (SD 0.14).

**Discussion:**

The most actionable priorities are related to the respect for their lands and customs, educational initiatives in schools about the importance of indigenous peoples to society, guarantees for basic health rights, and culturally appropriate provision of care, with specific mention of mental healthcare. The findings aligned closely with the concept of indigenous self-determination, which is rooted in autonomy and respect for cultural diversity, and the right to make decisions that impact their lives, land, and resources.

## 1. Introduction

Climate change has had a significant impact on indigenous people in Brazil. Brazil is home to ~900,000 indigenous peoples belonging to about 305 ethnic groups (1). Rising temperatures and changing rainfall patterns have led to crop failures and food insecurity, making it increasingly difficult for communities to maintain traditional livelihoods such as hunting and fishing (2–4). As with global indigenous populations, connection to nature is central to their daily practices and beliefs. The environment is closely tied to the identity of indigenous communities, and this connection is reflected in their traditional practices, customs, and beliefs (5). For many indigenous communities, the land is not just a physical place but also a source of identity, history, and spiritual wellbeing.

Reciprocity is an essential principle in indigenous cultures; it refers to mutual energy exchange between humans and the natural world. This manifests in the form of traditional practices such as ritual offerings or ceremonies that are performed to maintain balance between humans and the environment. The principle is rooted in the belief that all living beings, including humans, are interconnected and interdependent (5, 6). A lack of reciprocity impacts the ability to engage in traditional practices, disrupts cultural continuity, and widens indigenous health disparities (7). The most pressing challenge relates to deforestation, which also impacts the rainforests' crucial role in stabilizing the global climate and indigenous survival. The report released by the Intergovernmental Panel on Climate Change (IPCC), titled “Climate Change 2021: The Physical Science Basis”, highlighted that man-made changes are irrefutable and will worsen if we do not take practical actions to change the narrative of the climatic, environmental, and societal crises (8).

International organizations have increasingly recognized indigenous people's rights to health and wellbeing (9–11). The United Nations Declaration on the Rights of Indigenous Peoples (UNDRIP) provides an international framework to ensure indigenous populations' survival, dignity, and wellbeing worldwide (11). Many of the sustainable development goals (SDGs) are relevant to the welfare of indigenous people, particularly those that relate to addressing health and socio-economic inequalities. Reference is made to indigenous people (though only 6 times) in the SDGs, in the targets under Goal 2 on Zero Hunger and Goal 4 on education (12). In 2017, the World Health Organization published “Policy on Ethnicity and Health” (13), which highlighted strengthening the institutional and community capacity to generate evidence for policy-making to address the inequalities in health experienced by indigenous peoples. The report by the Pan American Health Organization (PAHO) “Health Plan for Indigenous Youth” identified several priority areas, including access to intercultural health services, traditional medicines, mental health, disabilities, and violence (14).

Young indigenous peoples are increasingly contributing to global discussion forums. They had a strong presence in the 27th Conference of the Parties of the United Nations Framework Convention on Climate Change. Despite this historic step change in participation, protecting indigenous peoples in the Global Goal on Adaptation was weak (15). Exclusion in decision-making promotes feelings of powerlessness, which links to a disproportionate burden of poor health (15, 16). Findings from the Xunati Uti study in Mato Grosso do Sul, Brazil showed that indigenous adolescents perceived their health and happiness to be influenced by their ecosystems, family life, friendships, nature, and belonging to a strong community (17). In a national survey in Brazil on violence against youths (2011–2017), only 1% of the sample was indigenous (*n* = 3,467), but the findings showed a higher likelihood of physical (71.8 vs. 63.3%) and sexual (29.8 vs. 21.3%) violence compared with White Brazilian youths (18). There are also concerns over high rates of substance use and suicides and that deforestation, climate-related disasters, and disproportionate impacts of COVID-19 have exacerbated mental health problems, however, mental healthcare is limited (19, 20). Expenditure on mental health in Latin America is generally inadequate (~2% of total public expenditure on health), particularly for community care, as ~61% is disbursed to inpatient psychiatric care (21).

The current study used a mixed-methods approach of concept mapping (CM) to identify what indigenous university students perceived to be important and feasible actions to protect the survival of indigenous peoples from climate change-related impacts (22). Concept mapping prioritizes stakeholder engagement at every stage of the research process, aligning well with principles of indigenous self-determination and gearing the focus of the research toward potentially translational learning for policymakers, implementors, and communities (23).

## 2. Methods

### 2.1. Ethics and recruitment

Ethical approval for this study was obtained from the National Research Ethics Commission (CONEP), protocol CAAE 36372820.0.0000.8027 from May 25th, 2021. All students signed an agreement form.

One researcher at UEMS (Universidade Estadual de Mato Grosso do Sul) and one researcher at UFRGS (Universidade Federal de Rio Grande do Sul) advertised the study among indigenous students. Concept mapping activities took place between August and September 2021.

This was a convenience sample and students attended the university of the authors (AJG and IMAVD). Each year, four indigenous youths are offered a scholarship to undertake undergraduate courses (for example, medicine, nursing, and psychology). A total of 20 indigenous university students were invited to participate in the study, and all agreed to take part.

### 2.2. Study design

#### 2.2.1. Concept mapping

Four meetings were conducted virtually through Google Meet, each lasting between 30 min and 2 h. Participatory CM is a structured process that generates statements from the discourse of students, which are later sorted and rated for importance and feasibility (24). The key steps include (1) brainstorming in response to a focus prompt, (2) sorting and rating, and (3) map interpretation sessions. All steps were conducted in a virtual environment. Each session was facilitated by researchers.

#### 2.2.2. Brainstorming

The first meeting was held to explain the objective of the research and obtain participant consent. Following consent, students completed an online demographic questionnaire. The brainstorming activity was guided by the focus prompt:

“*To protect the survival of the Indigenous Peoples from climate change it is necessary to…*”

Each student was asked to write their statements on paper during the brainstorming session and send statements via email to the facilitating researcher. In total, 104 statements were generated. Researchers removed repeated statements and, in discussion with the students, amended some statements for clarity. The final list included 46 statements.

#### 2.2.3. Sorting and rating

Each student was invited to organize the 46 statements into clusters based on what they perceived to be conceptually similar using an online Google Form. Students were then asked to rate each statement on the Google Form according to their perception of importance and feasibility, using a 5-point Likert scale. The questions read “*How important are each of the following statements regarding climate change and the adaptation of Indigenous People to it?”* and “*How feasible is it to implement each of the following statements into practice?”* The 5-point rating scales were: 1 = Relatively unimportant, 2 = Somewhat important, 3 = Moderately important, 4 = Very important, 5 = Extremely important; and for the feasibility of achieving a positive change: 1 = Not at all feasible, 2 = Somewhat feasible, 3 = Moderately feasible, 4 = Very feasible, 5 = Extremely feasible.

A third session was held to discuss the multidimensional maps with students to agree on appropriate clusters.

#### 2.2.4. Feedback session with students

A fourth session was held to discuss the results with students and ensure their validation of the results. The students were encouraged to make any changes they felt necessary to improve the representation of the results. They discussed the 8-cluster map and agreed it was the best representation of the key ideas.

### 2.3. Data analysis

Quantitative multivariate analyses of clusters and ratings were conducted to produce multiple visual maps of perceived actionable priorities. These analyses used the Group Wisdom TM software (22). All data collected through emails and Google forms were entered manually by two researchers and double-checked. First, a matrix of similarities was generated to check the statements and the labels given to each group of statements. Second, multidimensional scaling (MDS) analysis was then used to create a two-dimensional “point map”. Each statement was represented as a numbered point, with points closest together more conceptually similar. The stress value of the point map is a measure of how well the MDS solution maps the original data, indicating a good fit. Stress values range from 0 to 1, with lower values indicating better fit. The acceptable range for stress values is 0.205–0.365 (25). Finally, hierarchical cluster analysis was used to delineate clusters of statements (points) that were conceptually similar to create cluster maps based on the positioning of the statements on the point map. Cluster labels were determined by the indigenous students. Clusters with low bridging values (BV) indicate high agreement among students in the clustering of statements. Go-zone graphs showed the most actionable (high importance and high feasibility) and least actionable (low importance and low feasibility) statements (24).

## 3. Results

### 3.1. Profile of participating students

Twenty indigenous university students from Mato Grosso do Sul and Rio Grande do Sul states participated in all steps; 60% were women (*n* = 12) and 90% were not married (*n* = 18). Half of the stakeholders were of Kaingang ethnicity (*n* = 10) and received a university stipend (*n* = 13), and 60% considered themselves as rural indigenous (*n* = 12) (from a rural area).

Participant sociodemographic characteristics are presented in Table 1.

**Table 1:** Demographic characteristics of participating students.

**Age**	**Mean**	***N***	**%**
	25.25 y (SD4.63)		
Gender	Male	8	40
	Female	12	60
Marital status	Not married	18	90
	Married	2	10
Ethnicity	Kaingang	10	50
	Pitaguary	1	5
	Terena	3	15
	Atikum	4	20
	Arapium	1	5
	Tabajara	1	5
Income support	Scholarship	13	65
	Parents help	3	15
	None	4	20
Urban–rural residence before university	Urban	8	40
	Rural	12	60

### 3.2. Cluster map

**Figure 1:**
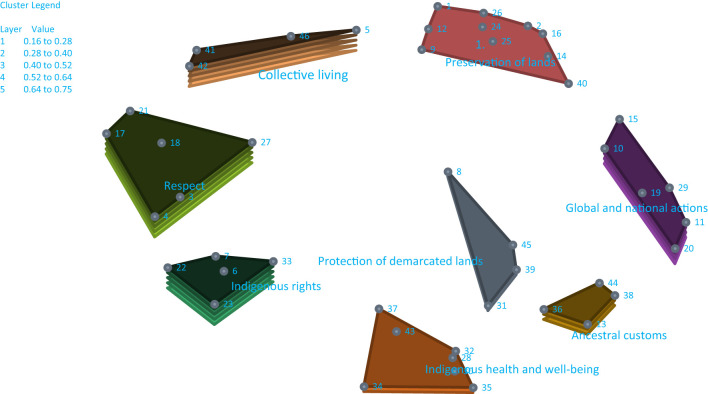
Cluster concept map showing the factors that indigenous university students felt could influence the protection of the survival of the indigenous peoples from climate change.

Statements and bridging values for each statement and cluster are presented in Table 2. The statements were assigned to 8 clusters: preservation of lands 0.16 (SD 0.13), respect 0.75 (SD 0.14), collective living 0.71 (SD 0.21), indigenous rights 0.64 (SD 0.23), protection of demarcated lands 0.31 (SD 0.10), ancestral customs 0.46 (SD 0.04), global and national actions 0.61 (SD 0.13), and indigenous health and wellbeing 0.35 (SD 0.14). Figure 1 shows the 8-cluster map. The layers of each cluster reflect the degree of agreement across students in the clustering of statements, with a few layers representing higher correspondence in clustering across the students. The highest agreement among students in the clustering of statements was for the following clusters: **preservation of lands; protection of demarcated lands; ancestral customs; and indigenous health and wellbeing**.

**Table 2:** Clusters with their statements: bridging values and importance and feasibility ratings (standard deviation).

**Cluster**		**Statement**	**Bridging value mean (SD)**	**Importance mean (SD)**	**Feasibility mean (SD)**
**Preservation of lands (*****n*** = **10–21.75%)**			0.16 (0.13)	4.56 (0.68)	3.77 (1.07)
	2	Stop deforestation, river pollution, and land depletion	0.01	4.65 (0.67)	3.7 (1.12)
	1	To preserve the environment, we live in	0.02	4.8 (0.52)	3.85 (0.93)
	16	Urgently prevent human-caused pollution and deforestation	0.07	4.6 (0.79)	3.95 (1.22)
	25	Ensure the preservation or restoration of soil quality	0.13	4.55 (0.69)	3.9 (1.12)
	9	Awareness, appreciation, and preservation of nature	0.15	4.65 (0.59)	3.85 (1.08)
	26	Ensure the preservation or recovery of biodiversity	0.15	4.5 (0.76)	3.7 (1.12)
	24	Ensure the *preservation or recovery of springs* and others	0.16	4.5 (0.60)	3.75 (1.05)
	14	There is sustainable development	0.20	4.55 (0.77)	3.7 (1.15)
	12	Learn new sustainable ways to use natural resources	0.28	4.2 (0.85)	3.55 (1.14)
	40	Rescue of deforested areas, using native species, carried out by the indigenous	0.49	4.8 (0.61)	3.85 (1.19)
**Protection of demarcated lands (*****n*** = **4–8.70%)**			0.31 (0.10)	4.58 (0.71)	3.86 (1.23)
	31	Prevent making a profit from the cost of indigenous life	0.20	4.55 (0.76)	3.85 (1.31)
	39	Ensure and monitor compliance with laws	0.26	4.5 (0.69)	3.75 (1.21)
	45	New land demarcation	0.34	4.55 (0.83)	3.85 (1.27)
	8	Demarcation of indigenous territories for the preservation of the environment	0.46	4.7 (0.57)	4.0 (1.21)
**Indigenous health and wellbeing**			0.35 (0.14)	4.42 (0.71)	3.98 (1.09)
	32	Promote actions for greater equity in the care given to indigenous peoples	0.08	4.3 (0.73)	3.85 (1.04)
	28	Promote basic sanitation in indigenous communities	0.30	4.6 (0.68)	3.85 (1.23)
	35	Greater assistance for the health of people who live isolated by choice	0.34	4.45 (0.69)	3.95 (1.19)
	37	Promote equity, considering the differences and particularities of each community	0.35	4.45 (0.69)	3.85 (1.08)
	43	Educational initiatives in schools about indigenous peoples and their importance in society	0.38	4.5 (0.51)	4.35 (1.04)
	30	Creation of artisanal wells in the villages through the municipal government, together with FUNAI (National Foundation for indigenous people)	0.43	4.2 (0.95)	3.9 (1.07)
	34	Assistance for mental health care	0.58	4.5 (0.69)	4.1 (1.02)
**Ancestral customs (*****n*** = **6–13.04%)**			0.46 (0.04)	4.55 (0.71)	3.78 (1.14)
	44	Create laws that respect the ancestry and territoriality of indigenous peoples	0.39	4.5 (0.69)	3.75 (1.16)
	36	Guarantee the basic health rights of the indigenous population, considering social, cultural and language aspects	0.46	4.65 (0.59)	4.00 (1.07)
	38	Change in laws for the protection and rights of indigenous peoples	0.48	4.45 (0.83)	3.65 (1.31)
	13	Implement policies that help guarantee the exclusive rights of indigenous peoples to lands already demarcated	0.51	4.6 (0.75)	3.7 (1.03)
**Global and national actions (*****n*** = **6–13.04%)**			0.61 (0.13)	4.45 (0.71)	3.63 (1.24)
	19	Implement environmental protection laws	0.40	4.45 (0.69)	3.9 (1.25)
	10	Review of global guidelines related to the environment	0.48	4.3 (0.86)	3.55 (1.14)
	11	Taxation of countries that negatively interfere with environmental changes	0.59	4.25 (0.79)	3.2 (1.40)
	20	Government to prevent clandestine mining in indigenous lands	0.66	4.7 (0.47)	3.8 (1.20)
	15	Respect environmental legislation	0.75	4.4 (0.75)	3.55 (1.23)
	29	Receive public resources, whether technological, material, financial and human for the preservation and recovery of the environment	0.75	4.6 (0.60)	3.75 (1.21)
**Indigenous rights (*****n*** = **5–10.90%)**			0.64 (0.23)	4.53 (0.67)	3.87 (1.17)
	33	Respect indigenous peoples and their struggle	0.33	4.10 (0.83)	3.90 (0.79)
	7	Respect the rights of indigenous peoples	0.52	4.55 (0.69)	4.05 (1.23)
	6	Fight with us so that the rights of indigenous peoples are respected	0.53	4.60 (0.50)	3.80 (1.23)
	22	Ensure the livelihoods of indigenous peoples	0.84	4.50 (0.83)	3.75 (1.06)
	23	Ensure the right to ancestral territory	0.97	4.55 (0.76)	3.80 (1.09)
**Collective living (*****n*** = **4–8.70%)**			0.71	4.15 (0.94)	3.53 (1.24)
	5	Respect the life of the environment	0.36	4.60 (0.68)	3.95 (1.05)
	41	Mankind come to love nature as the mother who bears him fruit	0.77	4.00 (0.92)	3.1 (1.29)
	42	Live collectively, knowing that one depends on the other and that everyone depends on nature	0.84	4.15 (0.93)	3.25 (1.12)
	46	Integrate indigenous knowledge to address environmental changes caused by climate change and land scarcity	0.87	3.85 (1.09)	3.8 (1.36)
**Respect (*****n*** = **6–13.04%)**			0.75 (0.14)	4.36 (0.79)	3.75 (1.09)
	27	Recognize and understand indigenous Peoples as stewards of the earth	0.59	4.25 (0.79)	3.75 (1.12)
	3	Respect the territory of indigenous Peoples	0.64	4.8 (0.41)	4.00 (1.02)
	4	Respect sacred territory	0.67	4.65 (0.59)	4.05 (1.05)
	21	Understand that the environment is sacred	0.76	4.10 (0.91)	3.4 (1.14)
	18	Ensure medicinal herbs	0.87	4.20 (0.89)	3.65 (1.09)
	17	Ensure traditional food	1	4.15 (0.81)	3.65 (1.08)

#### 3.2.1. Preservation of lands, protection of demarcated lands, ancestral customs, and indigenous health and wellbeing

The cluster **Preservation of lands** had a BV of 0.16 (SD 0.13) and 10 statements with a mean rating for importance of 4.6 (SD 0.68) and feasibility of 3.77 (SD 1.07). The students' statements highlighted the urgency for nature preservation, stopping deforestation and river pollution, and recovering biodiversity. The cluster **Protection of Demarcated Lands** had a BV of 0.31 (SD 0.10) and 4 statements with a mean rating for importance of 4.58 (SD 0.71) and feasibility of 3.86 (SD 1.23). The students' statements focused on the enforcement of the law to disallow the use of their lands for profit. Although **Ancestral customs** had a BV of 0.46, (SD 0.04); mean ratings for importance 4.55 (SD 0.71) and feasibility 3.78 (SD 1.14)] formed a separate cluster, the emphasis was similar. The 4 statements emphasized the urgency of implementing laws for land protection. The cluster **indigenous health and wellbeing** had a BV of 0.35 (SD 0.14) and seven statements with a mean rating for importance of 4.42 (SD 0.71) and feasibility of 3.98 (SD 1.09). The statements focused on the need for basic sanitation, clean potable water, and safe disposal of human waste in their villages, educational initiatives in schools about indigenous peoples and their importance in society, and access to healthcare (particularly mental healthcare).

#### 3.2.2. Global and national actions, indigenous rights, collective living, and respect

The high BVs for these clusters reflected variations in the clustering of statements across students, but the ratings of the statements reflected moderate importance and feasibility. **Global and national actions** [BV 0.61 (SD 0.13); mean ratings for importance 4.45 (0.71) and feasibility 3.63 (1.24)] highlighted the need for a review of global guidelines related to the environment, taxation to prevent countries implementing actions that have negative environmental impacts, and for preventing illegal activity (e.g., mining) which degrades the land and pollutes the water. **Indigenous rights** [BV of 0.64 (0.23); importance 4.53 (0.67) and feasibility 3.87 (1.17)] highlighted the need for systems to ensure the demarcation of their lands which they depend on for their livelihoods. **Collective living** [BV 0.71 (0.21); importance 4.15 (0.94) and feasibility 3.53 (1.24)] highlighted the loss of natural habitat and land scarcity. **Respect** [BV 0.75 (0.14); importance 4.36 (0.79) and feasibility 3.75 (1.09)] highlighted respect for indigenous peoples as stewards of the earth and for their cultural habits. The statements reflect overlapping meanings which explains the lack of close correspondence across the students in clustering.

### 3.3. Go zone map

**Figure 2:**
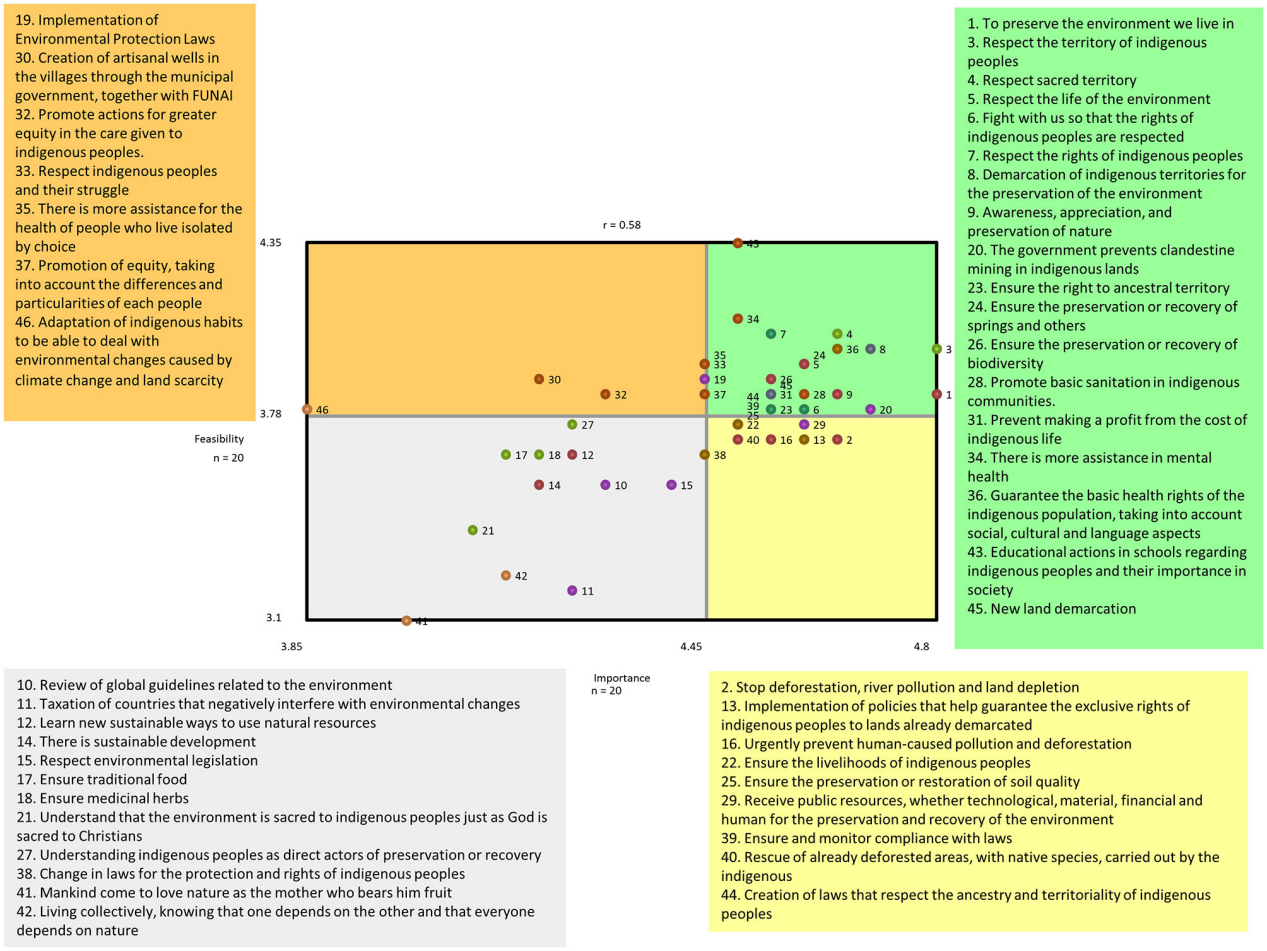
Go Zone map showing mean ratings for importance and feasibility for each statement. The colors of each point illustrate which cluster the statements belong to.

Go map zone is presented in Figure 2. The Go Zone map gives a visual representation of actionable priorities generated from the statements. The upper right-hand quadrant represents the statements that were rated most important and feasible to implement. The most highly rated statements on both importance and feasibility (mean ratings of ≥4) are related to the demarcation of and respect for their lands, educational initiatives in schools about indigenous peoples, and having a guarantee for basic health rights that considers their culture. Statements that were reported as least important and feasible are those in the lower left-hand quadrant. Examples of these statements included a “review of global guidelines related to the environment” and “live collectively, knowing that one depends on the other and that everyone depends on nature”.

## 4. Discussion

### 4.1. Principal findings

University students identified eight clusters that reflected the key factors that influence the survival of indigenous peoples in the context of climate change—preservation of lands, protection of demarcated lands, indigenous health and wellbeing, ancestral customs, global and national actions, indigenous rights, collective living, and respect. The most actionable priorities are related to the respect for their lands and customs, educational initiatives in schools about the importance of indigenous peoples to society, guarantees for basic health rights, and culturally appropriate provision of care. These findings align closely with the concept of indigenous self-determination, which is rooted in autonomy and respect for cultural diversity, and the inherent right to make decisions that impact their lives, land, and resources. Self-determination is critical to upholding human rights, social justice, and reconciliation, fostering partnerships based on mutual respect, and enabling indigenous peoples to contribute to local, national, and global development agendas while safeguarding their rights, lands, and identities (26).

#### 4.1.1. Respect, culture, and the role of young people

The students articulated the need for respect regarding their indigenous knowledge and relationships with nature which affects their health and wellbeing (14, 15, 25). Due to climate change impacts, many are unable to fully observe traditions tied to ancestral lands and this enforced inobservance can cause adverse impacts to health and wellbeing. Students discussed at length the need to understand that the earth is a source of indigenous identity, which is inextricably linked to the overall state of health and wellbeing of indigenous communities (1, 8, 27). Indigenous and Western scholarship alike has advocated for the integration and respect of indigenous worldviews, to link up diverse pathways of knowing and to address environmental events brought upon by climate change and wider disparities (1, 8, 27). The statements illustrated the gravity of the underrepresentation of indigenous voices in legal and political matters. They also reflected a strong perception of the need for systemic actions to address indigenous–non-indigenous inequalities to sustain and protect indigenous rights. The participatory exercise of CM created an inclusive space to advocate for a response to safeguard their communities. While indigenous young people are some of the most vulnerable people globally and are often disproportionately affected by political decisions, they are excluded from decision-making processes (14, 15, 27). The inclusion of young people is an important catalyst in policy and program advancement, as it offers an intergenerational perspective to support present and future generations vulnerable to the immediate and distal impacts of climate change (28–31).

#### 4.1.2. Brazilian context

The Brazilian Federal Constitution (1988) recognizes indigenous possession of the land as original, that is, before the creation of the Brazilian Federation (32). Legislation such as “*Marco temporal das terras Indígenas”*, however, has made this challenging as it encouraged territorial and land disputes, caused social and economic instability, and promoted violence against indigenous peoples on their native lands (33). Brazil has seen slow progress toward the implementation of legislation that promotes and protects the rights of indigenous peoples and their lands (34–36). This issue dominated many of the statements from participants as they felt there was an urgent need for action (5, 13). While government action and global and national actions were rated highly important, participants were less likely to rate the actions as feasible. This is likely to reflect the doubts acquired from the slow progression and the political climate created by former President Jair Bolsonaro at the time of this study (37). To safeguard their communities, indigenous peoples have been encouraging the implementation of alternative means associated with technological innovations that integrate traditional knowledge and enhance the capacities of pro-environmental and indigenous-oriented organizations (38). There has been some progress on this through funding bodies, such as the Brazilian Amazon indigenous-Podaali, that aim to promote the implementation of socio-environmental policies (34). However, efforts from multisectoral stakeholders to achieve system-wide changes remain patchy (35).

#### 4.1.3. Considerations for international conversations

The need for implementation of international legislation for environmental protection was a prominent topic in the students' discussions. Globally, indigenous peoples fight to protect their ancestral lands and to mitigate harmful impacts of climate change (1, 12, 28, 35, 36, 39). They are at the center of many discussions on the impacts of climate change and are considered the most responsible communities in environmental preservation (14, 15). According to *Complicity in Destruction IV: How mining companies and international investors drive Indigenous rights violations and threaten the future of the Amazon*, over the last 5 years, miners received a total of US$ 54.1 billion in financing from American, Brazilian, and other international investors. Additionally, US$ 14.8 billion was invested in research applications overlapping indigenous lands (40). There was stark awareness in the students' discussion of the global impact of the destruction of the Amazon Forest by influential international stakeholders. They discussed the Amazon as a vital source of biodiversity and the role it plays in regulating the earth's climate. Its destruction is a significant contributor to greenhouse gas emissions that drive climate change (35). Land restitution is being/or has been addressed in some parts of the globe. For example, in Canada, First Nation communities have regained the rights of a portion of the boreal forest east of Lake Winnipeg, one of the world's most intact ecosystems. Along with the provincial and national governments, the First Nations asked UNESCO to recognize the 29,000 km^2^ of Pimachiowin Aki or “The land that gives life”, as a World Heritage Site, to protect and restore the health of the land and those who rely on it (5, 41). A positive step forward is the international effort to address the legacies of Bolsonaro's reign. The new Brazilian President Luiz Inacio Lula da Silva and governments from other South American countries (Colombia, Guyana, Bolivia, Venezuela, Suriname, Peru, and Ecuador) that share the Amazon forest have recently declared their shared interest in protecting the forest from further exploitation at an Amazon forest summit in August 2023.

### 4.2. Strengths and limitations

While there was 100% retention of students throughout each stage of the concept mapping process, a larger and more diverse sample size representing more ethnicities from different villages and young people who are not at university would have enriched the interpretative value. A key strength of the study was the use of concept mapping which is a participatory method. Indigenous students generated the ideas and agreed on priorities. The visual conceptualization of ideas appealed to them and encouraged the discussions on the survival of their communities. Championing young indigenous voices is critical for charting the path to the sustainable development of interventions for system-wide changes.

### 4.3. Future directions

Indigenous health and wellbeing was the important factor identified in the context of climate change and the survival of indigenous peoples. Global epidemiological research has shown a widening of health inequalities between indigenous and non-indigenous populations. The divide is illustrated by shorter life expectancies at birth, persistent chronic disease, higher rates of sexually transmitted disease, maternal and infant mortality, and teen pregnancies, compared to non-indigenous (16). Students discussed the need for improving access to healthcare, particularly in relation to mental health, with an emphasis on intercultural models of care. In Brazil, there is emerging support for indigenous-specific health system reform, with an emphasis on holistic perspectives and stakeholder participation (39). National policies in Brazil, such as the National Policy for the Care of Indigenous Peoples, aim to address the poor socioeconomic conditions among indigenous communities and increase access to primary care (40). The policy has, however, been criticized for failing to integrate indigenous traditional knowledge (17). In comparison with Australia and New Zealand, there is less engagement of indigenous peoples in the development of intercultural health programs in Brazil. Services for indigenous communities in Brazil are often provided by non-indigenous visiting practitioners. Relocation to urban centers for employment is also common due to the lack of capacity of indigenous communities. The Brazilian Institute of Geography and Statistics (IBGE) reported that in the census of 2010, 49% of the total population of Brazilian indigenous lived in urban centers, outside demarcated indigenous lands (1).

## 5. Conclusion

The study aimed to capture the perspectives of indigenous university students on climate change and the survival of indigenous peoples in Brazil. They identified the key factors as preservation of lands, protection of demarcated lands, indigenous health and wellbeing, ancestral customs, global and national actions, indigenous rights, collective living, and respect. The students shared important and feasible changes that can be implemented to safeguard their communities. The knowledge from this study underpins a recently funded study (led by the authors) that will co-develop interventions with multisectoral partners to protect the health and wellbeing of indigenous youths in Brazil. The continuity of community–academic partnerships and capability building of young indigenous researchers are important considerations in indigenous research.

## Data availability statement

The raw data supporting the conclusions of this article will be made available by the authors, without undue reservation.

## Ethics statement

This study was approved by National Research Ethics Commission (CONEP), protocol CAAE 36372820.0.0000.8027 from May 25th, 2021. The studies were conducted in accordance with the local legislation and institutional requirements. The participants provided their written informed consent to participate in this study.

## Author contributions

AG, SH, PJ, ID, and AV coordinated the study, edited, and revised the manuscript. JS, MR, and LB analyzed the quantitative data and wrote the first draft of the manuscript with additions from XZ and LR. All authors were involved in designing the study. All authors contributed to the article and approved the submitted version.
